# Evidence-Based Medicine: Feminist Criticisms and Implications for Women's Health

**DOI:** 10.1089/whr.2022.0032

**Published:** 2022-10-20

**Authors:** Lea Merone, Komla Tsey, Darren Russell, Andrew Daltry, Cate Nagle

**Affiliations:** ^1^ College of Healthcare Sciences, James Cook University, Townsville, Australia.; ^2^ College of Arts, Society and Education, James Cook University, Smithfield, Australia.; ^3^ Cairns Sexual Health Service, Cairns North, Australia.; ^4^ Cairns and Hinterland Hospital and Health Service, Cairns, Australia.

**Keywords:** evidence-based medicine, feminism, feminist epistemologies, philosophy of medicine, women's health

## Abstract

Evidence-based medicine (EBM) dates back to 19th-century Paris and started out as a new paradigm for practicing medicine, with the aim of replacing anecdote with high-quality evidence from positivist-style research. Despite the clear logic underpinning EBM, there have been numerous criticisms, including maintenance of an archaic view of evidence as “facts,” failure to acknowledge that all research is underpinned by the beliefs of the researcher, and the simple fact that medical research has historically been androcentric and results generalized to female patients. In this essay, we discuss the criticisms of EBM, with a focus on feminist critiques based on three central feminist epistemologies: feminist empiricism, standpoint theory, and social constructivism. We argue that EBM potentially perpetuates gaps in women's health and advocate for incorporating feminist epistemologies into future medical research to garner further understanding of social influences on women's health. In addition, we argue that EBM may degrade the clinical acumen and that critical thinking should become a key component of medical school curricula.

“To question the foundations of a discipline or practice is not necessarily to deny its value, but rather to stimulate a judicious and balanced appraisal of its merits.”^1^

Evidence-based medicine (EBM) started out as a new paradigm for practicing medicine, replacing theoretical reasoning and anecdote with evidence from high-quality studies.^2^ From the outset, critics have expressed concerns that methods for gathering evidence are flawed and questioned the external validity of studies gathering information from groups of people and applying them to the individual.^3^ In this study, we will review the conceptual background of EBM, discuss some of the general criticisms of EBM, and finally critique EBM from a feminist perspective.

## EBM: Setting the Scene

The philosophical origins of EBM date back to 19th-century Paris and became mainstream in the medical communities in the 1980s and 1990s.^4^ By the mid-1990s both undergraduate and postgraduate medical programs had incorporated EBM into their curricula. EBM is defined as the explicit use of the current best evidence in deciding the clinical care of the individual patient.^4^ Traditionally, practicing EBM requires combining clinical acumen and expertise with external evidence based on high-quality clinical research.^4,5^ Clinical acumen is important, particularly when considering the differing social and economic circumstances of individual patients.

The best available evidence is defined as clinically relevant human research surrounding diagnostic tests and the efficacy of treatments.^6^ Neither clinical acumen nor best evidence alone is enough for safe patient care and the practice of effective safe clinical medicine is underpinned by both.^4^ External evidence should strongly inform, but never replace, clinical acumen. Clinical expertise should be utilized to determine if the best available evidence applies to the individual circumstances of the patient and how it should be integrated into individual patient care.^7^

EBM developed in response to poorly designed observational research that rendered the clinician dependent on personal professional expertise.^8^ Alongside it came refreshed enthusiasm for positivist-style science; the application of the scientific method that operationalizes, measures, and analyzes characteristics to detect patterns in covariation,^9,10^ and the EBM movement attempts to remove more intuitive aspects from clinical medicine and replace it with more vigorous scientific approaches.^10^ The EBM movement is centered on five interlinked ideas^10^:

1. Clinical decisions must be based on the best available evidence.
2. The clinical problem should determine the type of evidence sought.
3. Identifying best evidence should be through epidemiological and statistical thinking.
4. Evidence-based conclusions are only useful if put into practice.
5. Performance should be consistently evaluated.

**FIG. 1.**
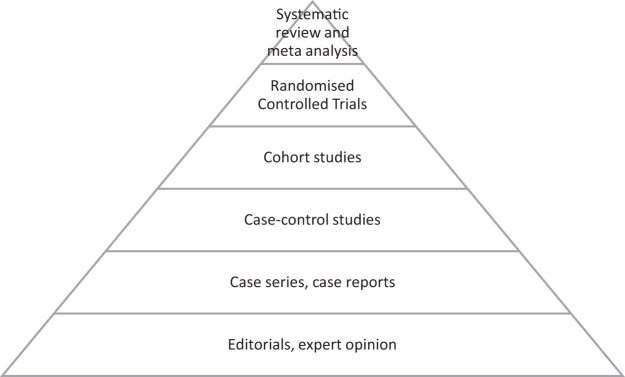
The hierarchy of evidence.^9^

The centerpiece of EBM is the hierarchy of evidence (Fig. 1),^11^ which places meta-analysis and systematic review at the top of pyramid, as the strongest form of evidence, followed by randomized controlled trials (RCTs), with opinion pieces at the base of the pyramid.

## Criticisms of EBM

Despite the clear logic underpinning EBM, there have been numerous criticisms from a variety of researchers both within and outside of the medical sphere. Goldenberg argues that EBM is problematic in that it maintains archaic views of evidence as “facts.”^10^ Positivism has been undermined by post-positivist philosophies of science^10^ as flawed in that the only positions it acknowledges as meaningful are those that are measurable and, therefore, scientifically verifiable.

Philosopher Kuhn claimed that our observations are in fact underpinned by our background beliefs and assumptions, be they consciously or unconsciously, and, therefore, can never truly be objective.^12^ In addition, Hume argued that our observations are always the product of interpretation.^10,13^ The Duhem–Quine thesis states that it is impossible to test one single scientific theory in isolation because empirical testing will always require one or more background assumptions that several other hypotheses or measurements are correct.^14^ Quine described this further as underdetermination, whereby every scientific theory will have at least one opposing theory that is supported by the scientific evidence.^15^

Underdetermination tells us that the evidence available to us at any given time may be insufficient to determine what conclusions we should draw in response to it.^14^ Chin-Yee^16^ argues that EBM exacerbates underdetermination because there are a number of auxiliary hypotheses in clinical trial settings. These auxiliary hypotheses are propagated by confounders found in real-life settings such as social and environmental factors that cannot be controlled (compared with laboratory settings where control enables fewer alternative explanations for a given result).^16^

EBM is based on evidential hierarchies that ultimately underdetermine the core beliefs of medical practice, resulting, according to Chin-Yee, in an “epistemic attitude that is skeptical of disease pathology,” limiting medical research by neglecting theoretical frameworks that may better integrate knowledge.^16^ Chin-Yee argues that the emphasis EBM places on the RCT devalues other valid epistemologies and underdetermines clinical medicine and the knowledge base used for clinical practice.^16^ Feminist social epistemologists have characterized underdetermination as a “gap” between theory and observation. Facts only provide evidential support for a theory in conjunction with an auxiliary hypothesis. Two researchers with differing background assumptions may legitimately interpret evidence in different ways.^17^

Guidelines and protocols are derived from EBM resources.^18^ In terms of clinical practice, critics of EBM describe concerns of the impact of guidelines and protocols on the clinical acumen of the doctor. Degradation of clinical expertise and reliance on medical tests and technology^19,20^ can create confusion when presented with the “atypical” patient who does not fit the standard diagnostic criteria^21^ nor respond to treatment as expected. Mant states “a clinical trial is the best way to assess whether an intervention works but it is arguably the worst way to assess who will benefit from it.”^22,23^ Population differences (genetic, cultural, and health systems) are different from individual patient differences (comorbidity, age, and previous treatments) making translation from large RCT data to the individual all the more challenging; a concept known as the ecological fallacy.^22^

RCTs often do not recruit any patient who is outside of the “average” for that disease,^24^ and inclusion criteria are strict.^22^ In addition, the conditions generated in RCTs, with high-tech laboratories, meticulous follow-up, and strict inclusion criteria, are often quite far removed from the conditions encountered in real-life clinical practice.^22^ Also, “average” patients are often defined from clinical studies that have historically excluded women and thus women may not be included in the typical patient picture.^25^ Recent cross-sectional analysis of published clinical trials in Australia has demonstrated that in certain specialties, this sex and gender gap in research lingers and may continue to obscure the clinical picture for females and people of other genders.^26^

EBM is often used to create clinical patient care protocols, defined as a diagnosis-specific written statement of standard procedures for clinical care against which clinicians can be assessed and their practice standardized.^27^ Protocol-driven medicine has many criticisms, including giving clinicians a ceiling of knowledge and care, limiting expertise, and clinical acumen.^28^ Limited expertise can have adverse effects when presented with the “atypical” patient. Furthermore, EBM has been criticized as increasing reliance on technology, erasing the human aspect and reducing patients to “technological objects.”^29,30^ Medical humanists express concern that EBM does not take adequately into account the patient preference or choice.

One challenge of practicing EBM is the time required for clinicians to keep pace with the latest evidence.^4^ In addition, there is a noted 17-year lag between publication of evidence and translation of findings into clinical practice, resulting in clinicians reliance on data that are potentially out of date.^31^ Combined with the only relatively recent calls to require the inclusion of women in clinical research,^32,33^ this could significantly adversely impact the health of female patients, particularly considering implementation of including women in clinical research has been slow.^34^

## Androcentricity and EBM

Historically, medical research has been conducted on the male body and the results broadly generalized to women^35^ and the health of those who identify as intersex, transgender, or other genders.^36^ As a construct of the man-made world, medicine is androcentric,^25^ not only assuming male bodies to be the norm, but also regarding male-dominated knowledge as the most valid.^37^ Since ancient philosopher Aristotle determined women's biology to be that of a “mutilated male,”^38^ women's bodies have been deemed too biologically erratic to be useful or valuable in scientific study.^37^

Androcentrism assumes that all people are valued according to male standards. The androcentrism of medical research can perpetuate stereotypes of women as “difficult” when they do not respond to treatments as expected (as per the male norm). Historically, women's health problems have been attributed to either their reproductive organs or their mental health,^37^ and these myths are observed in modern medicine; women are more likely than men to be discharged during serious medical events,^39^ and to have their physical symptoms attributed to mental illnesses.^40,41^

In addition to the inherent flaws of EBM, it is stipulated as part of the process that continuous methodological evaluation is required for a true EBM process to occur; however, there is little evidence that this is occurring, particularly with regard to women's health, with many trials failing to recruit adequate numbers of female participants and where women are recruited, results are often not analyzed by sex or gender.^26^ This means that an EBM approach fails women's health on many levels.

There is evidence that incorporating EBM into undergraduate courses enhances medical student critical thinking^42^; however, there is also evidence that EBM degrades the ability of the clinician to think critically. Factors that contributed to this inhibition include continuing medical education courses, pharmaceutical industry updates, physician experience, role models, and published reviews of health care practices.^43^

One of the largest contributors, however, is the development of clinical practice guidelines, which clinicians are largely expected to adhere to. There is emerging evidence that clinicians who choose to think rather than follow guidelines have a greater chance of experiencing litigation,^43^ thereby discouraging use of clinical acumen. EBM encourages practice based on the “average” or “typical” patient; however, individuals, as noted, rarely present in standard manners in real-life settings. It is, therefore, arguable that EBM inhibits critical thinking when presented with the “atypical” patient, which may contribute to the gender gap and misdiagnoses observed for female patients.

## EBM and Feminist Epistemologies

Consequently, from the feminist perspective, EBM is problematic. We can examine this from three feminist epistemologies: feminist empiricism, feminist standpoint, and social constructivism.

Feminist empiricism aligns with the foundation of EBM in that it argues for realism; there is a truth and reality to be discovered that researchers can objectively observe and study. Sexism and androcentrism may be managed and removed with the application of rigorous scientific methods. This epistemology allows feminists to work within current scientific paradigms by advocating for “better” science.^44^ Feminist empiricists conduct positivist science; assuming there is an objective reality to discover.^44^ In this way, feminist empiricists could bridge the gap between EBM advocates and critics, contextualizing empirical evidence on the grounds that no theory develops in isolation.^17,45^

Empiricist feminists argue that strict methodological controls as seen in EBM and the RCT cannot (and must not) filter out the social background of all involved in the research. They advocate for more authentic accounts of the interconnections between knowledge and socioeconomic and political relations.^17^ These researchers utilize standard positivist methodology with an underlying awareness of the sex and gender biases that underpin research.^46^ Feminist empiricists typically utilize one of two themes: that production of knowledge is a social process or that communities (as opposed to individuals) are the agents of knowledge.^17^

Feminist standpoint theorists argue that positivist approaches to science do not fully acknowledge the influence of social context and prior experience on the researcher and their processes for developing, conducting, and analyzing research.^44^ Indeed, for EBM, there is a notable lack of acknowledgement of the social background of patients and the impact of the experiences of the researchers on their results and interpretation. The perspective of feminist standpoint theorists is that women's experiences have not been represented effectively in research because they are framed in patriarchal concepts, language, and perspectives.^44^

Standpoint feminists postulate that the scientific method itself is responsible for scientific accounts that echo the patriarchal social relations and influences.^47^ They hypothesize that scientific method is lacking true objectivity and serves the desires of (largely) the men^48^ who conduct it**.** Consequently, their argument is that there is need for a new scientific method that does not reject attributes considered to be “feminine,” such as emotions and perceptions.^47^ Standpoint feminist, Evelyn Fox Keller, believed that science needed to be de-gendered and involve acknowledgement of feelings and intuitions.^47^

Social constructivists, or postmodernists,^49^ argue that science creates reality rather than reflects it; therefore, researchers are an inherent component of knowledge construction. Social constructivist theory posits that seeking an objective truth is not possible; truths are relative and dependent on the social context of the researcher and the subjects. This allows for the idea of multiple truths and realities located in time, place, and person (known as pluralism).^44^ In contrast to feminist standpoint theory, feminist social constructivists suggest there are many conflicting social discourses and none should be privileged; however, they acknowledge that under current paradigms, a power-neutral knowledge does not exist.^50^ Social constructivism emphasizes the individual's experience of the world, rejecting empiricisms ideals of objectivity.

The way language shapes experience is a fundamental part of social constructivism, known as discourse, “a series of statements which construct an object.”^51^ Discourse refers to meanings, metaphors, representations, and stories that cohere to produce particular versions of events. Language creates reality.^44,52^ Social constructivists focus on multiple discourses and reject the idea of “woman” as a category because it is socially constructed and exclusionary to those who are members of other oppressed groups outside of the white cis-heterosexual middle-class women: women of color, women of different socioeconomic groups, transgender women, those assigned female at birth and identify as nonbinary or another gender, and those with different sexual orientations.^50^ This is challenging in the EBM sphere, which frequently groups people enrolled in clinical trials by categories such as sex and gender. Despite this categorization, results are often not analyzed by sex nor gender,^26^ rendering this subcategorization moot.

## EBM: Moving to an Inclusive Future

From the feminist epistemology, there are many gaps observable in EBM and these may contribute to some of the discrepancies between the care of women and men observed in clinical practice and outcomes. For EBM to move forward and assist in narrowing the sex and gender gap in clinical medicine, trials need to address their systemic failings and acknowledge underdetermination. Incorporating the feminist epistemologies and stronger understanding of societal influences into future scientific study and clinical practice can only support and enhance EBM for the benefit of both the patient and the clinician.

Feminist empiricism, standpoint, and social constructivism all advocate for use of multiple sources of knowledge and from the feminist perspective, the RCT alone is insufficient to provide evidence for clinical practice. Utilizing the results from other study designs, such as observational studies, alongside the RCT may reduce underdetermination by incorporating evidence that is gathered in more natural settings. Allowing an expansion on the definition of EBM to include well-conducted observational studies higher up the hierarchy of evidence and alongside the RCT may enhance the evidence base for women's health.

Finally, the clinical acumen must not be degraded more, but rather supported by EBM and utilized alongside the current best evidence.^53^ Critical thinking should be reinforced within the medical school curricula to enable doctors to assess the evidence while holding their experience in esteem, particularly when considering that no patient is *typical*.

## Abbreviations Used

EBM: evidence-based medicine
RCT: randomized controlled trial
